# Polyphasic taxonomic description of Streptomyces okerensis sp. nov. and Streptomyces stoeckheimensis sp. nov. and their biotechnological potential

**DOI:** 10.1099/ijsem.0.006716

**Published:** 2025-03-13

**Authors:** Imen Nouioui, Eveline Derr, Alina Zimmermann, Marlen Jando, Gabriele Pötter, Sarah Kirstein, Meina Neumann-Schaal, Cathrin Spröer, Boyke Bunk, Yvonne Mast

**Affiliations:** 1 German Collection of Microorganisms and Cell Cultures, Leibniz Institute DSMZ, Inhoffenstraße 7B, 38124 Braunschweig, Germany; 2 Rhenish Friedrich Wilhelm University of Bonn, Regina-Pacis-Weg 3, 53113 Bonn, Germany; 3 Institute of Pharmaceutical Biology and Biotechnology, Heinrich Heine University Düsseldorf, Universitätsstraße 1, 40225 Düsseldorf, Germany; 4 Braunschweig Integrated Centre of Systems Biology, Rebenring 56, 38106 Braunschweig, Germany; 5 Institut für Mikrobiologie, Technische Universität Braunschweig, Rebenring 56, 38106 Braunschweig, Germany

**Keywords:** *Actinomycetota*, diversity, ecology, systematics

## Abstract

*Streptomyces* strains DSM 116494^T^ and DSM 116496^T^ were isolated from sediment samples of the River Oker in Braunschweig, Germany, and subjected to a polyphasic taxonomic study and genome mining for specialized secondary metabolites. Phenotypic, genetic and genomic data confirmed the assignment of these strains to the *Streptomyces* genus. Pairwise 16S rRNA gene sequence similarity values between the strains and validly named *Streptomyces* species reached 99.5 and 99.7% for strains DSM 116494^T^ and DSM 116496^T^, respectively. Genome-based phylogeny demonstrated that *Streptomyces pilosus* and *Streptomyces griseoflavus* species were the close relatives to strain DSM 116494^T^, while *Streptomyces vinaceus* species was the nearest neighbour to strain DSM 116496^T^. Digital DNA–DNA hybridization and average nucleotide identity comparisons of the genomic sequence of the strains and their close phylogenomic relatives revealed that values were below the determined threshold of 70 and 95–96% for prokaryotic species demarcation, respectively. The strains were distinguished from their close neighbours based on biochemical, chemotaxonomic and enzymatic data. Given these results, the strains merit being affiliated to novel species within the genus *Streptomyces*, for which the names *Streptomyces okerensis* sp. nov. (=OG2.3^T^=DSM 116494^T^=KCTC 59408^T^) and *Streptomyces stoeckheimensis* sp. nov. (=OG3.14^T^=DSM 116496^T^=KCTC 59410^T^) are proposed. Strains DSM 116494^T^ and DSM 116496^T^ harboured several biosynthetic gene clusters encoding potentially novel antimicrobial and anticancer compounds. Crude extracts of strains DSM 116494^T^ and DSM 116496^T^ inhibited the growth of Gram-negative bacteria (*Escherichia coli* ΔtolC, *Proteus vulgaris*) and a multi-drug-resistant Gram-positive, *Staphylococcus aureus*.

## Introduction

Life-threatening infections caused by multi-resistant bacteria are a serious public health problem that calls for more investigation into natural product (NP) discovery, as synthetic antibiotics are burdened by high costs and long procedures, with a low success rate. Previous studies have shown that bacteria of the phylum *Actinomycetota* are a great source of antimicrobial and anticancer bioactive compounds, two-thirds of which are clinically relevant [1]. Several actinobacterial taxa are considered gifted micro-organisms due to their large genome size accompanied by an extensive genetic potential to produce specialized secondary metabolites, such as the genus *Streptomyces* [2]. The latter belongs to the family *Streptomycetaceae* [3] and contains more than 730 species validly named [4] (https://lpsn.dsmz.de/genus/streptomyces), with *Streptomyces albus* as the type species [3,5]. *Streptomyces* strains are characterized by their filamentous cell structure with a substrate mycelium followed by the development of aerial mycelium and spores. These bacteria are known for their saprophytic lifestyle and have been found in different ecological habitats, including extreme environments. The chemotaxonomic features of this taxon are as follows: cell wall peptidoglycan with ll-diaminopimelic acid (DAP; ll-A_2_pm); diphosphatidylglycerol (DPG), phosphatidylethanolamine (PE), phosphatidylinositol (PI) and phosphatidylinositol mannosides as major polar lipids; MK-9(H_6_) and MK-9(H_8_) as the predominant quinones; iso and anteiso saturated fatty acids [6]. The genus has undergone several taxonomic revisions and amendments as a result of the modernization of prokaryotic systematics [7,13]. Advances in genomic sequence technology and bioinformatics have significantly improved the taxonomic status of several taxa and the NP discovery strategy based on taxogenomics and genome mining approaches. Previous studies have proved that bioprospecting for novel bioactive compounds is especially promising when focusing on the novelty of biological material at a taxonomic and/or ecological level, as it is known that phylogenetically unique strains tend to produce novel biochemistry [14,16]. Hundreds of antibiotics and biomolecules of significant interest to industry and pharmaceuticals have been produced by *Streptomyces*, making this taxon a promising source of novel bioactive NPs [1,17,19]. Exploiting the genetic diversity of this taxon is of interest to a broad scientific community, including microbiologists, taxonomists, (bio)chemists and pharmacists working on NP discovery. In this context, a selective isolation campaign of novel *Streptomyces* strains from an unexploited habitat, such as the river Oker in Lower Saxony (Germany), was carried out. Little is known about the microbial diversity at this site, which contains sediments from the Rammelsberg mining site. Two *Streptomyces* isolates, OG2.3^T^ (=DSM 116494^T^) and OG3.14 ^T^ (=DSM 116496^T^), isolated from sediment collected from the Oker River, Braunschweig, Germany, were subjected to polyphasic taxonomic studies, and their biotechnological and pharmaceutical potential was assessed using a genome mining approach and *in vitro* antimicrobial bioassays. Isolates OG2.3^T^ and OG3.14^T^ were found to form novel *Streptomyces* species for which the names *Streptomyces okerensis* sp. nov. and *Streptomyces stoeckheimensis* sp. nov. are proposed. Both isolates harbour diverse and unique biosynthetic gene clusters (BGCs) potentially encoding novel chemical entities.

## Isolation, habitat and maintenance

Isolates OG2.3^T^ (=DSM 116494^T^) and OG3.14^T^ (=DSM 116496^T^) were recovered from a sediment sample collected from the Oker River, Braunschweig, Germany (52° 12′ 35.6″ N 10° 31′ 9.7″ E), and deposited at the Leibniz Institute DSMZ-German Collection of Microorganisms and Cell Cultures and the Korean Collection for Type Cultures (KCTC) under culture collection accession numbers DSM 116494^T^=KCTC 59408^T^ and DSM 116496^T^=KCTC 59410^T^, respectively. In brief, 1 g of sediment was subjected to pre-treatment using 3% SDS and 4% NaOH for decontamination as described by Parashar *et al*. [20]. The treated sediments were suspended in 1 ml of saline solution (0.9%, w/v) followed by serial dilution (10^−1^, 10^−2^, 10^−3^, 10^−4^, 10^−5^). Selective agar media, DSMZ 65, yeast extract–malt extract agar (ISP2) and starch casein agar supplemented with nalidixic acid (25 µg ml^−1^) and cycloheximide (25 µg ml^−1^) were inoculated with 100 µl of diluted samples. After incubation at 28 °C for 14 days, isolates OG2.3^T^ (=DSM 116494^T^) and OG3.14^T^ (=DSM 116496^T^) were transferred to medium DSMZ 65, and a pure culture was obtained after two sub-culturing. The optimal growth conditions for isolates OG2.3^T^ (=DSM 116494^T^) and OG3.14^T^ (=DSM 116496^T^) are available in the DSMZ public catalogue (https://www.dsmz.de/collection/catalogue). The reference strains, *Streptomyces pilosus* DSM 40097^T^, *Streptomyces griseoflavus* DSM 40456^T^ and *Streptomyces vinaceus* DSM 40515^T^, were obtained from the DSMZ culture collection (https://www.dsmz.de/collection/catalogue).

## Morphological and growth properties

The growth properties of the isolates were determined after testing their ability to grow in the presence of a wide range of agar media [International *Streptomyces* Project (ISP), ISP1 (DSMZ 1764), ISP2 (DSMZ 987), ISP3 (DSMZ 84), ISP4 (DSMZ 252), ISP5 (DSMZ 993), ISP6 (DSMZ 1269), ISP7 (DSMZ 1619), glucose–yeast extract–malt extract (GYM)=DSMZ 65, Trypticase soy agar (TSA)=DSMZ 535, N-Z amine (DSMZ 554), Czapek peptone agar (DSMZ 83), temperature (4, 10, 15, 25, 28, 37, 42 and 45 °C), pH (5, 5.5, 6, 6.5, 7, 7.5, 8, 8.5, 9) and NaCl (2.5, 5, 7.5, 10%)]. The bacterial suspension used for these tests was prepared from 7-day-old cultures of the isolates in ISP2 medium incubated at 28 °C. The density of the inoculum was equivalent to 5 on the McFarland scale [21]. Purity of the culture was examined using a light microscope (Carl Zeiss; West Germany). Morphological features of the isolates regarding the colour of the substrate and aerial mycelia were determined using the RAL colour chart. The growth properties tests were carried out in triplicates.

Isolates DSM 116494^T^ and DSM 116496^T^ showed a morphological and microscopic cell structure consistent with the genus *Streptomyces* [6]. Both isolates exhibited branched substrate and aerial mycelia on ISP3, ISP4 and GYM agar plates. Isolate DSM 116494^T^ grew well on ISP1, ISP2, ISP4, ISP6, GYM, TSA and Czapek peptone agar plates, where aerial mycelium of different colours was observed (Table S1, available in the online Supplementary Material). The type strains of *S. pilosus* DSM 40097^T^ and *S. griseoflavus* DSM 40456^T^ formed grey aerial mycelium in the presence of GYM agar medium after 7 days incubation at 28 °C. Isolate DSM 116496^T^ had good growth, mainly on ISP3, ISP4 and GYM plates, where ivory aerial mycelium was developed after 7 days incubation at 28 °C (Table S1). *S. vinaceus* DSM 40515^T^ showed good growth on GYM and DSMZ 84 agar media and developed ivory-white aerial mycelium after 7 days incubation at 28 °C on GYM medium. Strains DSM 116494^T^ and DSM 116496^T^ were able to grow from 10 to 28 °C, at a pH of 5–9 and up to 2.5% NaCl. However, only isolate DSM 116494^T^ was able to grow at 37 °C and in the presence of up to 7.5% NaCl. The morphology of the above-mentioned strains is publicly available in the DSMZ online catalogue (https://www.dsmz.de/collection/catalogue/microorganisms/catalogue).

## Biochemical, enzymatic and chemotaxonomic features

The biomasses of the isolates and their close phylogenomic neighbours, harvested from 7-day-old cultures prepared in ISP2 at 28 °C, were washed three times with saline solution (0.9%) and then freeze-dried. The lyophilized cells were subjected to a chemotaxonomic analysis known to be of value for the genus *Streptomyces*. The DAP isomers of the peptidoglycan were determined as described by Schleifer and Kandler [22]. Two-dimensional TLC was used to identify the polar lipid pattern of the strains, following the protocol of Minnikin *et al*. [23]. Fresh biomasses of the strains and their close phylogenetic neighbours, *S. pilosus* DSM 40097^T^, *S. griseoflavus* DSM 40456^T^ and *S. vinaceus* DSM 40515^T^, were used for isoprenoid menaquinone and cellular fatty acid analyses. The fatty acids of the strains were extracted [24], analysed by GS coupled to a flame ionization detector (Agilent instrument, model 6890 N) and identified using a GC-MS run on an Agilent GC-MS 7000D instrument [25]. Afterwards, fatty acid methyl esters were derivatized with dimethyl sulphide to resolve the position of the single double bonds [26]. The menaquinone pattern of the strains was determined following the protocol of Schumann *et al*. [27] by HPLC coupled to a diode array detector and a high-resolution mass spectrometer.

The biochemical and enzymatic features of the isolates and their close relatives were determined using the API 50CH, API 20 NE and API ZYM kits in accordance with the manufacturer’s instructions, Biomérieux, France. The density of the bacterial suspension used for these tests was equivalent to 5 on the McFarland scale [21].

Isolate DSM 116494^T^ was distinguished from its close phylogenomic neighbours, *S. pilosus* DSM 40097^T^ and * S. griseoflavus* DSM 40456^T^, by its ability to metabolize methyl-*α*-d-glucopyranoside, amygdalin and d-saccharose and produce *ß*-galactosidase and *α*-mannosidase. Strain DSM 116496^T^ was differentiated from its close phylogenomic neighbour, *S. vinaceus* DSM 40515^T^, by its ability to metabolize l-arabinose, d-cellobiose, phenylacetic acid and potassium nitrate (Table 1). Whole cell hydrolysates of the strains and their close phylogenomic neighbours were rich in ll-DAP of the peptidoglycan. The isolates DSM 116494^T^ and DSM 116496^T^ had a similar polar lipid profile containing PI, PE, DPG, unidentified lipids (Ls), phospholipids (PLs), amino lipids (AL), glycophosphatidylinositol and glycophospholipid (GPL), as shown in Fig. S1. The reference strains had PI, PE and DPG with a minor variation in their polar lipid profiles (Fig. S1). The major fatty acids (>5%) of both isolates consisted of iso-C_15:0_, anteiso-C_15:0_, anteiso-C_17:0_, C_16:1_ *cis* 9, iso-C_16:0_ and C_16:0_. However, strain DSM 40097^T^ had in addition a low percentage of iso-C_17:1_ *cis* 9 and iso*-*C_17:0_, while strain DSM 40515^T^ had iso*-*C_17:0_ and C_16:1_ *cis* 9. Strain DSM 40456^T^ showed a fatty acid profile similar to that of strain DSM 116496^T^, but with a minor amount of iso-C_15:0_. The complete fatty acid profile of the studied strains is displayed in Table S2. The predominant menaquinones (>15%) of the isolate DSM 116494^T^ and its close relatives, *S. pilosus* DSM 40097^T^, were MK-9(H_4_) and MK-9(H_6_), while * S. griseoflavus* DSM 40456^T^ also had MK-9 and MK-9(H_2_). A similar quinone profile was obtained for isolate DSM 116496^T^, as for strain DSM 116494^T^, whereas its close relative additionally had MK-9(H_8_) (Table 1).

**Table 1.** Phenotypic and genomic features of the strains DSM 116494^T^ and DSM 116496^T^ and their close phylogenomic relatives

	Studied strain	Close phylogenetic relative		Studied strain	Close phylogenetic relative
	**Isolate DSM 116494^T^**	***S. griseoflavus* DSM 40456^T^**	***S. pilosus* DSM 40097^T^**	**Isolate DSM 116496^T^**	***S. vinaceus* DSM 40515^T^**
l-Arabinose	+	+	−	+	−
d-Ribose	+	+	+	−	−
d-Xylose	+	+	−	−	−
d-Galactose	+	(+)	−	−	+
d-Fructose	+	+	+	−	(+)
l-Rhamnose	+	+	+	−	−
Inositol	+	+	(+)	−	−
d-Mannitol	+	+	+	−	+
Methyl-*α*-d-mannopyranoside	(+)	−	−	−	−
Methyl-*α*-d-glucopyranoside	+	−	−	−	−
Amygdalin	+	−	−	−	−
Arbutin	(+)	+	−	(+)	+
Salicin	(+)	−	−	+	(+)
d-Cellobiose	+	+	+	+	−
d-Maltose	+	−	+	−	+
d-Lactose (bovine origin)	+	+	−	−	−
d-Saccharose (sucrose)	+	−	−	−	−
d-Trehalose	+	+	+	−	−
d-Turanose	(+)	−	−	−	−
d-Arabitol	+	+	+	−	−
Potassium nitrate	+	+	−	+	−
l-Arginine	−	+	−	−	−
Urea	−	+	−	+	+
4-Nitrophenyl-*β*-d-galactopyranoside	+	−	+	+	+
Adipic acid	(+)	−	−	−	−
Trisodium citrate	−	−	−	(+)	(+)
Phenylacetic acid	−	−	−	+	−
Lipase (C14)	(+)	(+)	(+)	−	−
Trypsin	−	(+)	−	−	+
*α*-Chymotrypsin	(+)	(+)	(+)	−	+
Acid phosphatase	+	−	+	+	+
*α*-Galactosidase	−	−	(+)	−	−
*β*-Galactosidase	+	−	−	−	+
*β*-Glucosidase	−	(+)	−	+	+
*N*-Acetyl-*β*-glucosaminidase	+	+	+	−	+
*α*-Mannosidase	+	−	−	−	−
**Chemotaxonomic features**					
Diaminopimelic acid (DAP)	ll-DAP	ll-DAP	ll-DAP	ll-DAP	ll-DAP
Polar lipid profile	PI, PE, DPG, PLs, AL, GPL	PI, PE, DPG, PAL, PL_1-2_, GPI, GPL	PI, PE, DPG, GPI, AL, Ls, PLs	PI, PE, DPG, PLs, AL_1-2_, GPL_1-2_	PI, PE, DPG, PLs, GPL
Predominant menaquinone (>15%)	MK-9(H_4_) and MK-9(H_6_)	MK-9, MK-9(H_2_), MK-9(H_4_), MK-9(H_6_)	MK-9(H_4_) and MK-9(H_6_)	MK-9(H_4_) and MK-9(H_6_)	MK-9(H_4_), MK-9(H_6_),MK-9(H_8_)
Fatty acids (>5%)	iso-C_15:0_, anteiso-C_15:0_, anteiso-C_17:0_, C_16:1_ *cis* 9, iso-C_16:0_ and C_16:0_	anteiso-C_15:0_, anteiso-C_17:0_, C_16:1_ *cis* 9, iso-C_16:0_ and C_16:0_	iso-C_15:0_, anteiso-C_15:0_, anteiso-C_17:0_, C_16:1_ *cis* 9, iso-C_16:0_, C_16:0_, C_17:1_ *cis* 9 and iso*-*C_17:0_	iso-C_15:0_, anteiso-C_15:0_, anteiso-C_17:0_, C_16:1_ *cis* 9, iso-C_16:0_ and C_16:0_	iso-C_15:0_, anteiso-C_15:0_, anteiso-C_17:0_, C_16:1_ *cis* 9, iso-C_16:0_, C_16:0_, iso*-*C_17:0_ and C_16:1_ *cis* 9
**Genomic features**					
Genome size (Mpb)	7.8	7.5	7.5	9.5	7.6
G+C content (mol%)	71.8	72.3	72.3	71.6	72.3
N50	7330 005	115 138	465 971	9 131 297	−
Number of coding sequences	7280	7177	7105	8903	7188
Number of RNAs	85	69	71	91	89
Genome accession number	CP169548–CP169549	NZ_BMUC00000000.1	NZ_BMTE00000000.1	CP169550–CP169554	CP023692.1

+, positive reaction; -−, negative reaction; (+), weak reaction.

All the strains showed positive reactions to: glycerol, d-glucose, *N*-acetylglucosamine, esculinaesculin/ferric citrate, starch (aAmidon), glycogen, gentiobiose, potassium gluconate, d-mannose (API 50 CH); *β*-glucosidase, gelatin, d-glucose, d-mannose, potassium gluconate, malic acid (API 20 NE); alkaline phosphatase, esterase (C4), esterase lipase (C8), leucine arylamidase, valine arylamidase, cystine arylamidase, naphthol-asAS-biBI-phosphohydrolase, *α*-glucosidase (API ZYM). All the strains showed negative reactions to: erythritol, d-arabinose, l-xylose, d-adonitol, methyl-*β*-d-xylopyranoside, l-sorbose, dulcitol, d-sorbitol, d-melibiose, inulin, d-melezitose, d-raffinose, xylitol, d-lyxose, d-tagatose, d-fucose, l-fucose, l-arabitol, potassium 2-ketogluconate, potassium 5-ketogluconate (API 50 CH); l-tryptophan, d-glucose fermentation, capric acid (API 20 NE); *β*-glucuronidase, *α*-fucosidase (API ZYM).DPG diphosphatidylglycerol; PE phosphatidylethanolamine; PI phosphatidylinositol; AL aminolipid; L lipid; GPI glycophosphatidylinositol; GPL glycophospholipid; PAL phosphoaminolipid; PL phospholipid.

ALaminolipidDPGdiphosphatidylglycerolGPIglycophosphatidylinositolGPLglycophospholipidLlipidPALphosphoaminolipidPEphosphatidylethanolaminePIphosphatidylinositolPLphospholipid

## 16S rRNA gene-based identification

Seven-day-old biomass harvested from isolate cultures, prepared in ISP2 medium incubated at 28 °C with shaking at 150 r.p.m., was subjected to genomic DNA extraction [28], which was used for PCR-mediated amplification of a 16S rRNA and whole-genome sequencing at the DSMZ microbial DNA service. Amplification and sequencing of the 16S rRNA gene were performed using Applied Biosystems 24-capillary system. Pairwise 16S rRNA gene sequence similarity between isolates and their close phylogenetic neighbours was determined using the EZBioCloud server (https://www.ezbiocloud.net/) [29], from which the validly named close species were retrieved. The almost complete 16S rRNA gene sequence of the isolates (>1400 bp) generated by PCR was aligned and compared with that extracted from the genome sequence to confirm their authenticity using the Basic Local Alignment Search Tool (blastn) available on the NCBI (National Center for Biotechnology Information) web server (https://blast.ncbi.nlm.nih.gov/Blast.cgi) [30,31]. A maximum likelihood (ML) phylogenetic tree based on the 16S rRNA gene sequences was inferred via the type strain genome server (TYGS; https://tygs.dsmz.de/) [32,33].

A near full-length 16S rRNA gene sequence of the strains DSM 116494^T^ (1533 bp) and DSM 116496^T^ (1531 bp) was aligned with the type strains of validly named *Streptomyces* species, available in the EZBioCloud database. The strain DSM 116494^T^ showed 99.5% 16S rRNA gene sequence similarity with *Streptomyces marokkonensis* AP1^T^, while strain DSM 116496^T^ shared 99.7% similarity with the type strains of *Streptomyces xanthophaeus*, *Streptomyces cirratus*, *Streptomyces nojiriensis*, *Streptomyces spororaveus*, *Streptomyces subrutilus*, *Streptomyces avidinii*, *S. vinaceus* and *Streptomyces lavendulae* subsp. *lavendulae* species (Table S3). The isolates and several *Streptomyces* species (Table S3) had 16S rRNA gene similarity values above the threshold of 98.65–98.7% for prokaryotic species delineation [34,35]. Isolates DSM 116494^T^ and DSM 116496^T^ had 16S rRNA gene sequence similarity values of 96.6%. In the ML phylogenetic tree (Fig. S2), isolate DSM 116494^T^ was placed in a poorly supported distinct branch loosely associated with a subclade housing type strains of *Streptomyces albaduncus* (99%) and *S. griseoflavus* (99.2%) and distant from *S. marokkonensis* AP1^T^ (99.5%). Isolate DSM 116496^T^ formed a subclade with the type strains of *S. avidinii* and *S. subrutilus* (99.7%) species. These later were placed in the same subcluster as *S. xanthophaeus*, *S. cirratus*, *S. nojiriensis*, *S. spororaveus*, *S. vinaceus* and *S. lavendulae* subsp. *lavendulae* species. However, the phylogenetic position of the isolates within the radiation of the genus *Streptomyces* was not well supported, as reflected by a low bootstrap value. These results indicated the low resolution of the 16S rRNA gene to differentiate closely related species.

## Genome-based phylogeny and comparative genomic analysis

The genome of the isolates was sequenced using PacBio Sequel II (Pacific Biosciences, Menlo Park, CA, USA). The library construction protocol and the software and programme used for the preparation of reads and assemblies were described by Mahmoud *et al*. [36]. Genome quality was validated using LongQC v.1.2.0c [37] and no error correction was carried out. The genome sequences of the isolates DSM 116494^T^ and DSM 116496^T^ were annotated using the Rapid Annotation using Subsystem Technology (RAST) platform [38, 39] and deposited in GenBank under accession numbers CP169548 and CP169550, respectively. Whole-genome-based phylogeny was inferred via TYGS (https://tygs.dsmz.de/) [32,33].

Isolate DSM 116494^T^ and its close relatives *S. griseoflavus* JCM 4479^T^ and *S. pilosus* JCM 4372^T^ had a genome size of 7.8, 7.5 and 7.5 Mb; G+C content of 71.8, 72.3 and 72.3 mol%; N50=7 330 005, 115 138 and 465 971; number of coding sequences 7280, 7177 and 7105; RNAs 85, 69 and 71, respectively. Isolate DSM 116494^T^ contained one linear plasmid with a size of 533.741 kb (accession number CP169549). Isolate DSM 116496^T^ and its close phylogenomic neighbour *S. vinaceus* had a genome size of 9.5 and 7.6 Mb, G+C content of 71.6 and 72.3 mol%, number of coding sequences 8903 and 7188 and RNAs 91 and 89, respectively (Table 1). Isolate DSM 116496^T^ contained three linear plasmids with a size of 221.220 (accession number CP169552), 130.481 (accession number CP169553) and 19.702 kb (accession number CP169554) and one circular plasmid with a size of 4.449 kb (accession number CP169551).

The genomic characteristics of these studied strains were consistent with those of the genus *Streptomyces*.

**Fig. 1.**
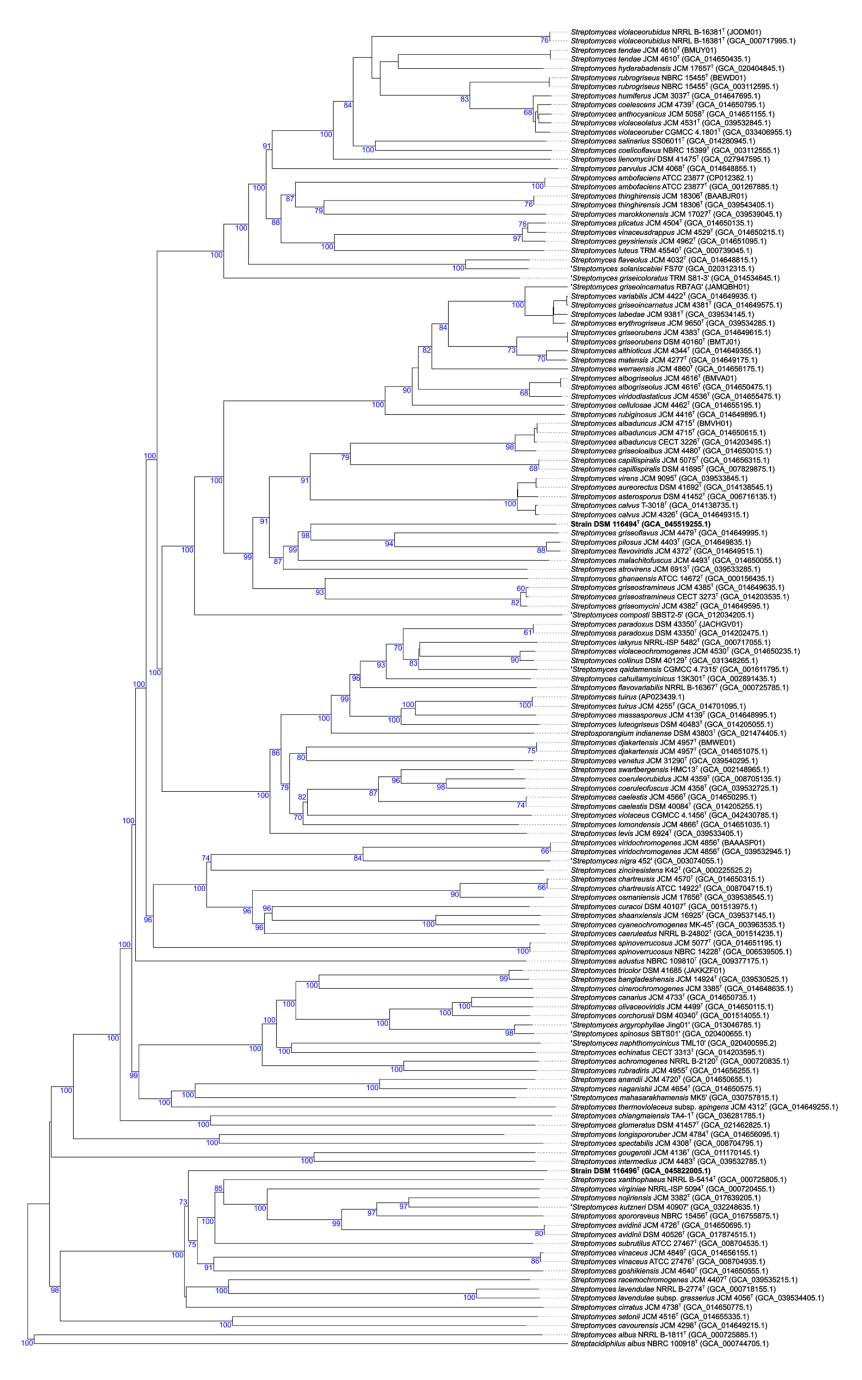
Genome-based phylogeny showing the phylogenetic relatedness of the strains DSM 116494^T^ and DSM 116496^T^ to their close neighbours of *Streptomyces* species validly named.

Genome-based phylogeny and comparative genomic approaches, digital DNA–DNA hybridization (dDDH) and average nucleotide identity (ANI), were conducted to provide the correct taxonomic species rank of the isolates and reliable data on their phylogenetic relatedness to validly named *Streptomyces* species. The 16S rRNA gene sequences extracted from the genome sequence of the isolates were identical to those derived by PCR, confirming the authenticity of strains DSM 116494^T^ and DSM 116496^T^. The phylogenomic tree showed that the isolate DSM 116494^T^ was placed in a distinct, well-supported branch, closely associated with a subclade encompassing type strains of *S. griseoflavus*, *S. pilosus* and *Streptomyces flavoviridis* (heterotypic synonym of *S. pilosus*) species, while *S. marokkonensis* appeared in another clade (Fig. 1). Isolate DSM 116496^T^ formed a well-supported divergent branch tightly related to a subcluster containing type strains of *S. xanthophaeus*, *Streptomyces virginiae*, *S. nojiriensis*, *S. spororaveus*, *Streptomyces kutzneri*, *S. subrutilus*, *S. avidinii*, *S. vinaceus* and *Streptomyces goshikiensis* species (Fig. 1). From these reference strains, *S. vinaceus* species is the most closely related to isolate DSM 116496^T^.

dDDH between the whole-genome sequence of isolates and their close phylogenomic relatives was estimated via the TYGS web server using the recommended formula d4 [33,40, 41]. ANI value between the genome sequence of the isolates and their close reference strains was generated by the ANI calculator tool available on the EZBioCloud server [42] (https://www.ezbiocloud.net/tools/ani). Genomic traits such as genome size, G+C content, number of coding sequences, number of RNA genes and N50 of the isolates were determined via the RAST server.

The dDDH values between the whole-genome sequence of isolate DSM 116496^T^ and its close phylogenomic neighbours were between 28.7 and 29.4%, values below the 70% cutoff point for prokaryotic species demarcation [43]. The dDDH value between isolate DSM 116496^T^ and its close neighbour *S. vinaceus* was 29.4% (Table S4). The dDDH values between the whole-genome sequence of isolate DSM 116494^T^ and its close relatives, *S. griseoflavus* (39%) and *S. pilosus* (38.5%), were lower than the determined borderline for the species designation listed above (Table S4). The dDDH value between the whole-genome sequence of isolates DSM 116494^T^ and DSM 116496^T^ was 22.3% (Table S4). These results were coherent with the ANI values between the genome sequence of the strains DSM 116494^T^ and DSM 116496^T^ and their close phylogenomic neighbours, * S. griseoflavus* JCM 4479^T^ (89.6%) and *S. pilosus* JCM 4372^T^ (89.5%) and *S. vinaceus* ATCC 27476^T^ (84.8%), which were inferior to the boundary for bacterial (95–96%) and *Streptomyces* (96.7%) species delineation, respectively [35,44,46]. Consequently, isolates DSM 116494^T^ and DSM 116496^T^ form new species within the evolutionary radiation of the genus *Streptomyces*, for which the names *S. okerensis* sp. nov. and *S. stoeckheimensis* sp. nov. are proposed, respectively.

## Antimicrobial bioassay and specialized secondary metabolites

The isolates were cultured in 50 ml of ISP2, NL19, NL800 and R5 50 media at 28 °C with shaking at 180 r.p.m. for 5 days at 28 °C as described by Nouioui *et al*. [47]. In summary, the crude extracts were obtained after mixing the bacterial cultures with ethyl acetate (v/v) for 6 h at room temperature, followed by drying of the organic phase using a centrifugal evaporator (SP Genevac EZ-2, ‘Low BP’ programme). Extracts were dissolved in methanol (0.25 ml) and used for antimicrobial testing, including Gram-positive (multi-resistant *Staphylococcus aureus* DSM 18827 and *Enterococcus faecium* DSM 20477^T^) and Gram-negative bacteria (*Escherichia coli* ΔtolC JW5503-1 and *Proteus vulgaris* DSM 2140), as well as yeast (*Candida albicans* DSM 1386). The detailed history and growth properties of these reference strains are available in the DSMZ online catalogue (https://www.dsmz.de/collection/catalogue). Antimicrobial tests were carried out as described by Nouioui *et al*. [47]. Three biological replicates were prepared.

Crude extracts of isolate DSM 116494^T^, generated from cultures prepared in R5 medium, inhibited the growth of *E. coli* ΔtolC and *S. aureus*. However, strain DSM 116496^T^ showed antibacterial activity only against *E. coli* ΔtolC, as shown in Table S5.

The whole-genome sequence of the strains was analysed with the antiSMASH v.7.0 web tool [48] for the abundance of BGCs encoding specialized secondary metabolites.

The complete genome sequence of the strains DSM 116494^T^ and DSM 116496^T^ contained BGCs associated with various secondary metabolite classes, such as clusters with genes encoding non-ribosomal peptide synthases, polyketide, polyketide synthase (PKS), terpene biosynthetic genes or genes encoding the biosynthesis of ribosomally synthesized and post-translationally modified peptides (RiPPs). These results highlight the genetic and metabolic ability of the strains to produce NPs. In total, the genome sequence of DSM 116494^T^ harboured 27 BGCs, of which 3 BGCs (lanthipeptide, RiPP-like and polyketide) are located on a plasmid, whereas DSM 116496^T^ contained 32 BGCs, of which 2 BGCs (butyrolactone and a large hybrid PKS–lanthipeptide BGC) are located on a plasmid (Fig. S3A, B). In total, 15 BGCs from strain DSM 116494^T^ and 23 BGCs from strain DSM 116496^T^ showed similarity values of <50% to known BGCs, indicating their potential to encode for novel NPs. Strain DSM 116494^T^ is characterized by a rather high content of terpene BGCs, whereas strain DSM 116496^T^ contained many BGCs encoding the biosynthesis of peptidic NPs (Table S6 and Fig. S3A, B).

The phenotypic, genetic and genomic data distinguish strains DSM 116494^T^ and DSM 116496^T^ from the *Streptomyces* species validly named and confirm their assignment to novel species for which the names *S. okerensis* sp. nov. and *S. stoeckheimensis* sp. nov. are proposed with strains DSM 116494^T^ and DSM 116496^T^ as type strains, respectively. *In vitro* and *in silico* screening for secondary metabolites and bioactive compounds underlined the biotechnological and pharmaceutical potential of these two proposed type strains. These results are in concordance with the hypothesis that new biology leads to new chemical entities [14,49] and emphasize the significant role of taxonomic studies in the selection of biological material for drug discovery.

## Description of *Streptomyces okerensis* sp. nov.

*Streptomyces okerensis* (o.ker.en’sis. N.L. masc. adj. *okerensis*, of Oker, the name of the river Oker, Braunschweig, Germany, from where the strain was isolated).

Gram-stain-positive, aerobic filamentous actinobacterium characterized by the presence of branched substrate and aerial mycelia which adopt different colours on ISP1 (agate grey aerial mycelium), ISP3 (dusty grey aerial mycelium), ISP4 (white greyish aerial mycelium), ISP6 (white aerial mycelium), GYM (dusty to platinum grey aerial mycelium), TSA (white grey aerial mycelium) and Czapek peptone (platinum grey aerial mycelium) agar plates. It is able to grow between 10 and 37 °C, at pH 5–9 and up to 7.5% NaCl. Optimal growth occurs on GYM, TSA and ISP6 media at 28 °C, pH 6.5–7.5. The strain is able to metabolize amygdalin, arbutin, d-arabitol, d-cellobiose, d-fructose, d-galactose, d-lactose (bovine origin), d-maltose, d-mannitol, d-mannose, d-ribose, d-saccharose (sucrose), d-trehalose, d-turanose, d-xylose, inositol, l-arabinose, l-rhamnose, methyl-*α*-d-glucopyranoside, methyl-*α*-d-mannopyranoside and salicin. It is able to reduce potassium nitrate and produce *β*-galactosidase (para-nitrophenyl-*β*-d-galactopyranosidase), alkaline phosphatase, esterase (C4), acid phosphatase, naphthol-AS-BI-phosphohydrolase, *α*-glucosidase, *N*-acetyl**-***β***-**glucosaminidase and *α*-mannosidase. Whole cell hydrolysates are rich in ll-DAP in the peptidoglycan. The predominant menaquinones (>15%) are MK-9(H_4_) and MK-9(H_6_). The major fatty acids are (>5%) iso-C_15:0_, anteiso-C_15:0_, anteiso-C_17:0_, C_16:1_ *cis* 9, iso-C_16:0_ and C_16:0_. Polar lipid profile consists of PI, PE, DPG, glycophospholipid, unidentified polar lipids and amino lipids. The genome size of strain DSM 116494^T^ is 7.8 Mb, with DNA G+C content of 71.8 mol%. The strain has one linear plasmid with a size of 533.741 kb (accession number CP169549).

The type strain DSM 116494^T^ (=OG2.3^T^=KCTC 59408^T^) was isolated from sediment collected from Oker River, Stöckheim, Braunschweig, Germany (52° 12′ 35.6″ N 10° 31′ 9.7″ E). The 16S rRNA gene and whole-genome sequences have been deposited in GenBank under accession numbers PQ657469 and CP169548, respectively.

## Description of *Streptomyces stoeckheimensis* sp. nov.

*Streptomyces stoeckheimensis* (stoeck.heim.en’sis N.L. masc. adj. *stoeckheimensis.*, of Stöckheim, the region from where the strain was isolated).

Gram-stain-positive, aerobic filamentous actinobacterium characterized by the presence of branched substrate and aerial mycelia which adopt different colours on ISP3 (light grey-ivory aerial mycelium), ISP4 (light ivory aerial mycelium) and GYM (warm ivory aerial mycelium) agar plates. It is able to grow between 10 and 28 °C, at pH 5–9 and up to 2.5% NaCl. Optimal growth occurs on GYM medium at 28 °C, pH 7–8. The strain is able to metabolize l-arabinose, d-mannose, arbutin, salicin, d-cellobiose, *N*-acetylglucosamine and phenylacetic acid; reduce potassium nitrate; produce urease, *β*-galactosidase, acid phosphatase and *β*-glucosidase. Whole cell hydrolysates are rich in ll-DAP in the peptidoglycan. The predominant menaquinones (>15%) are MK-9(H_4_) and MK-9(H_6_). The major fatty acids are (>5%) iso-C_15:0_, anteiso-C_15:0_, anteiso-C_17:0_, C_16:1_ *cis* 9, iso-C_16:0_ and C_16:0_. Polar lipid profile consists of PI, PE, DPG, glycophospholipid, unidentified polar lipids (PLs) and amino lipids (AL). The genome size of strain DSM 116496^T^ is 9.5 Mb, with DNA G+C content of 71.6 mol%. The strain has three linear plasmids with a size of 221.220 (accession number CP169552), 130.481 (accession number CP169553) and 19.702 kb (accession number CP169554) and one circular plasmid with a size of 4.449 kb (accession number CP169551).

The type strain DSM 116496^T^ (=OG3.14^T^=KCTC 59410^T^) was isolated from sediment collected from Oker River, Stöckheim, Braunschweig, Germany (52° 12′ 35.6″ N 10° 31′ 9.7″ E). The 16S rRNA gene and whole-genome sequences have been deposited in GenBank under accession numbers PQ657470 and CP169550, respectively.

## supplementary material

10.1099/ijsem.0.006716 Uncited Supplementary Material 1.

## Abbreviations

ANI: average nucleotide identity
BGCs: biosynthetic gene clusters
DAP: diaminopimelic acid
dDDH: digital DNA–DNA hybridization
DPG: diphosphatidylglycerol
GYM: glucose–yeast extract–malt extract
ISP: International *Streptomyces* Project
KCTC: Korean Collection for Type Cultures
ML: maximum likelihood
NCBI: National Center for Biotechnology Information
NP: natural product
PE: phosphatidylethanolamine
PI: phosphatidylinositol
PKS: polyketide synthase
RAST: Rapid Annotation using Subsystem Technology
RiPPs: ribosomally synthesized and post-translationally modified peptides
TSA: Trypticase soy agar
TYGS: type strain genome server
