# Supplementary material for: Polyphasic taxonomic description of Streptomyces okerensis sp. nov. and Streptomyces stoeckheimensis sp. nov. and their biotechnological potential
Source: Int J Syst Evol Microbiol. 2025 Mar 13;75(3):006716. doi: 10.1099/ijsem.0.006716 (PMC11936341; doi:10.1099/ijsem.0.006716)
Supplement: Uncited Supplementary Material 1. [file ijsem-75-06716-s001.pdf]

Supplementary Material

Polyphasic taxonomic description of *Streptomyces okerensis* sp. nov and *Streptomyces stoeckheimensis* sp. nov. and their pharmaceutical potential.

Imen Nouioui[1]*, Eveline Derr[1,2,3], Alina Zimmermann[1], Marlen Jando[1], Gabriele Pötter[1], Sarah Kirstein[1], Meina Neumann-Schaal[1,4], Cathrin Spröer[1], Boyke Bunk[1], Yvonne Mast[1,4,5]

[1]Leibniz-Institut DSMZ – German Collection of Microorganisms and Cell Cultures, Inhoffenstraße 7B, 38124 Braunschweig, Germany.

[2]Rheinische Friedrich-Wilhelms-University Bonn, Regina-Pacis-Weg 3, 53113 Bonn, Germany.

[3]Heinrich-Heine-University Düsseldorf, Institute of Pharmaceutical Biology and Biotechnology, Universitätsstraße 1, 40225 Düsseldorf, Germany

[4]Braunschweig Integrated Centre of Systems Biology (BRICS), Rebenring 56, 38106 Braunschweig, Germany.

[5]Technische Universität Braunschweig, Institut für Mikrobiologie, Rebenring 56, 38106 Braunschweig, Germany.

Corresponding author: Imen Nouioui, imen.nouioui@dsmz.de

Section: Actinobacteria

Supplementary Material

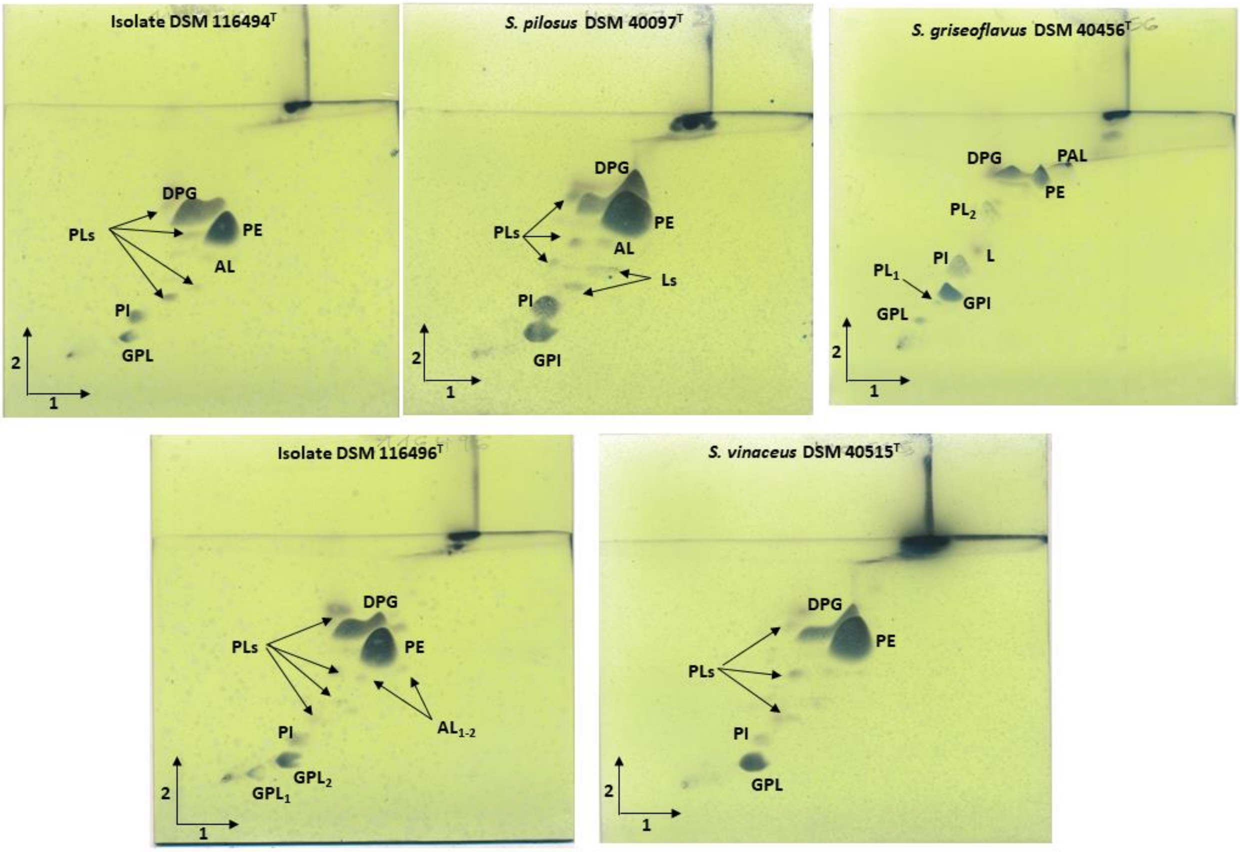

Figure S1. Two-dimensional TLC plate of polar lipids extracted from the strains DSM 116494^T and DSM 116496^T and their close phylogenomic relatives stained with molybdatophosphoric acid (SigmaP1518). Key: DPG diphosphatidylglycerol; PE phosphatidylethanolamine; PI phosphatidylinositol; AL aminolipid; L lipid; GPI glycophosphatidylinositol; GPL glycophospholipid; PAL phosphoaminolipid; PL phospholipid. Solvent1: chloroform: methanol: distilled water (65:25:4 v/v/v/); solvent 2: chloroform: glacial acetic acid: methanol: distilled water (80:12:15:4 v/v/v).

Supplementary Material

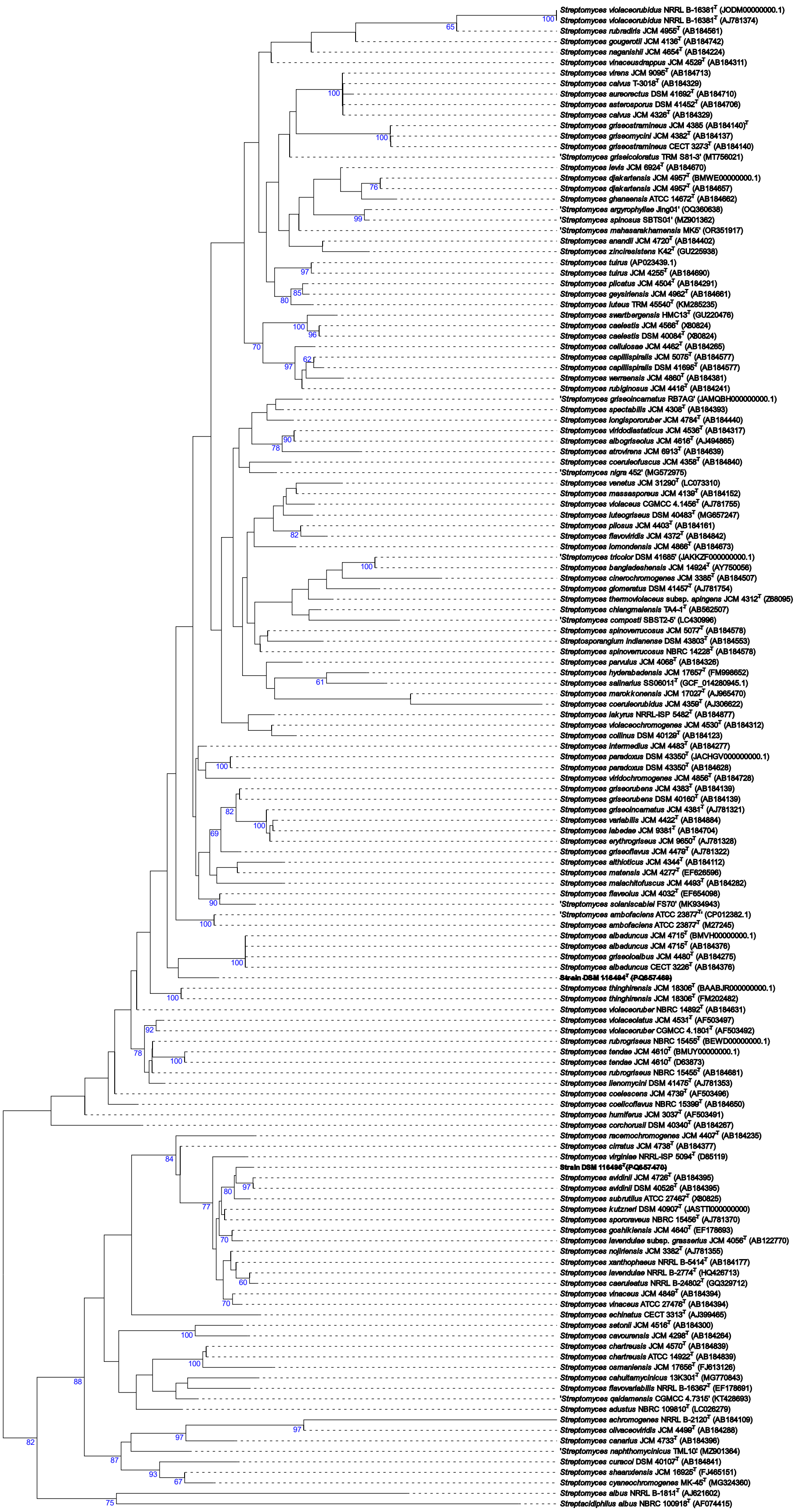

Supplementary Material

Figure S2. Maximum likelihood phylogenetic tree based on the 16S rRNA gene sequence showing the phylogenetic relatedness of the strains DSM 116494^T and DSM 116496^T to their closest neighbours of *Streptomyces* species validly named.

A

DSM 116494^T chromosome

Region	Type	From	To	Most similar known cluster		Similarity
Region 1.1	lanthipeptide-class-i, NRPS, lanthipeptide-class-ii	240,582	305,082	teicoplanin	NRP:Glycopeptide	5%
Region 1.2	resorcinol	409,384	450,520	lasalocid	Polyketide	11%
Region 1.3	NRP-metallophore, NRPS	527,565	584,393	peucechelin	NRP	50%
Region 1.4	T2PKS	866,870	939,379	spore pigment	Polyketide	83%
Region 1.5	ectoine	1,736,880	1,747,278	ectoine	Other	100%
Region 1.6	NRPS-like	2,461,588	2,505,667	streptozotocin	Other	23%
Region 1.7	terpene	2,809,981	2,831,810	desotamide	NRP	13%
Region 1.8	NI-siderophore	2,856,505	2,886,277	desferrioxamin B/desferrioxamine E	Other	100%
Region 1.9	butyrolactone	3,910,729	3,921,718			
Region 1.10	RRE-containing	5,049,461	5,070,534	oryzanaphthopyran A/oryzanaphthopyran B/oryzanaphthopyran C/oryzanthrone A/oryzanthrone B/ chlororyzanthrone A/chlororyzanthrone B	Polyketide	6%
Region 1.11	terpene	5,181,972	5,203,057	albaflavenone	Terpene	100%
Region 1.12	T3PKS, thiopeptide	5,274,196	5,349,991	granaticin	Polyketide:Type II polyketide	16%
Region 1.13	NI-siderophore	5,907,459	5,937,363	kinamycin	Polyketide	16%
Region 1.14	terpene	6,025,560	6,046,384			
Region 1.15	betalactone, T1PKS	6,163,977	6,208,535	meilingmycin	Polyketide	6%
Region 1.16	PKS-like, NRPS	6,243,118	6,317,279	cadaside A/cadaside B	NRP	19%
Region 1.17	T1PKS	6,345,539	6,392,912	enduracidin	NRP	33%
Region 1.18	RiPP-like	6,395,911	6,407,224			
Region 1.19	terpene	6,442,790	6,464,946	geosmin	Terpene	100%
Region 1.20	NI-siderophore	6,599,413	6,630,604	paulomycin	Other	11%
Region 1.21	terpene	6,635,381	6,661,735	isorenieratene	Terpene	100%
Region 1.22	hydrogen-cyanide, lanthipeptide-class-iii	6,960,166	6,990,137	aborycin	RiPP	64%
Region 1.23	terpene	7,045,688	7,072,457	hopene	Terpene	92%
Region 1.24	hydrogen-cyanide	7,245,253	7,258,087	aborycin	RiPP	14%

DSM 116494^T plasmid

Region	Type	From	To	Most similar known cluster		Similarity
Region 2.1	LAP, thiopeptide, RRE-containing	13,322	48,741	streptovaricin	Polyketide	17%
Region 2.2	RiPP-like	175,167	185,382	informatipeptin	RiPP:Lanthipeptide	42%
Region 2.3	NRPS-like, T1PKS	447,299	495,491	borrelidin	Polyketide	11%

B

Supplementary Material

DSM 116496^T chromosome

Region	Type	From	To	Most similar known cluster		Similarity
Region 1.1	NI-siderophore	524,323	555,713	A54145	NRP	6%
Region 1.2	melanin	635,426	665,346	melanin	Other	100%
Region 1.3	terpene	671,936	692,850	monensin	Polyketide	5%
Region 1.4	terpene , NRPS	741,140	803,363	azomycin	Other	83%
Region 1.5	CDPS	1,066,662	1,087,381	ibomycin	Polyketide	4%
Region 1.6	CDPS	1,230,194	1,250,934	tetronasin	Polyketide	3%
Region 1.7	thioamitides , thiopeptide	1,295,796	1,332,831			
Region 1.8	lanthipeptide-class-i	1,343,153	1,368,382			
Region 1.9	terpene	1,561,130	1,587,920	hopene	Terpene	61%
Region 1.10	lassopeptide , lanthipeptide-class-i	1,917,710	1,947,210	citrulassin B	RiPP	40%
Region 1.11	lanthipeptide-class-iv	1,988,906	2,011,881	corbomycin	NRP	7%
Region 1.12	terpene	2,034,960	2,057,176	geosmin	Terpene	100%
Region 1.13	RiPP-like	2,144,627	2,156,000			
Region 1.14	hydrogen-cyanide	2,226,071	2,239,280	aborycin	RiPP	21%
Region 1.15	NRPS , butyrolactone	2,274,768	2,368,737	coelichelin	NRP	100%
Region 1.16	T3PKS , NI-siderophore	2,448,987	2,520,358	kinamycin	Polyketide	30%
Region 1.17	CDPS	2,870,706	2,891,494			
Region 1.18	T3PKS , other , terpene	3,289,549	3,346,577	furaquinocin B	Terpene+Polyketide	39%
Region 1.19	NRPS , betalactone	3,454,301	3,530,085	enduracidin	NRP	20%
Region 1.20	phenazine	3,826,245	3,846,736	endophenazine A/endophenazine B	Other:Phenazine	38%
Region 1.21	butyrolactone	4,492,090	4,503,079	triacsin C	Other	6%
Region 1.22	NRPS	5,353,401	5,415,283	cinnapeptin	NRP	14%
Region 1.23	NAPAA	7,588,613	7,622,584	ε-Poly-L-lysine	NRP	100%
Region 1.24	NRPS-like	7,889,164	7,931,827	conglobatin	NRP	15%
Region 1.25	terpene	8,194,252	8,215,289			
Region 1.26	lanthipeptide-class-iii	8,322,475	8,345,102	SapB	RiPP:Lanthipeptide	100%
Region 1.27	ectoine	8,422,174	8,432,578	ectoine	Other	100%
Region 1.28	hglE-KS , T1PKS	8,452,718	8,505,891	hexacosalactone A	Other	6%
Region 1.29	T2PKS	8,593,777	8,666,283	spore pigment	Polyketide	66%
Region 1.30	T1PKS , lanthipeptide-class-i , NRPS , NAPAA	8,830,596	8,914,758	stenothricin	NRP:Cyclic depsipeptide	13%

DSM 116496^T plasmid

Region	Type	From	To	Most similar known cluster		Similarity
Region 3.1	butyrolactone	26,927	37,871	lactonamycin	Polyketide	3%
Region 3.2	lanthipeptide-class-iii , HR-T2PKS , thioamitides , arylpolyene , T2PKS , butyrolactone	76,959	221,220	prejadomycin/rabelomycin/gaudimycin C/gaudimycin D/ UWM6/gaudimycin A	Polyketide:Type II polyketide+Saccharide:Hybrid/ tailoring saccharide	43%

Figure S3. BGCs for strains DSM 116494^T (A) and DSM 116496^T (B) using AntiSMASH webserver.

Supplementary Material

Table S1. Growth properties of the isolates DSM 116494^T and DSM 116496^T in the presence of a wide range of agar media.

Strains	ISP-1	ISP-2	ISP-3	ISP-4	ISP-5	ISP-6	ISP-7	GYM	TSA	N-Z Amine	Czapek peptone
DSM 116494^T	+++ AM agate grey (RAL7038) / SM light orange brown (RAL8023)	+++ no AM/ SM maize yellow (RAL1006)	++ AM dusty grey (RAL7037) / SM brownish	+++ AM white - greyish / SM saffron yellow (RAL1017)	+++ no AM/ SM golden yellow (RAL1004)	+++ AM white / SM yellow orange (RAL2000) - orange brown (RAL8023)	+ no AM/ SM golden yellow (RAL1004)	+++ AM dusty to platinum grey / SM chrome yellow (RAL1007)	+++ AM white grey / SM chrome yellow (RAL1007) - yellow orange (RAL2000)	++ no AM/ SM saffron yellow (RAL1017)	+++ AM platinum grey (RAL7036) / SM ochre brown (RAL8001) - orange brown (RAL8023)
DSM 116496^T	+++ no AM / SM ochre yellow (RAL1024)	+++ no AM//SM light ivory (RAL 1015)	+++ AM light grey-ivory / SM ochre yellow (RAL1024)	+++ AM light ivory (RAL1015) / SM ochre yellow (RAL1024)	+++ no AM / SM ivory (RAL1014)	+++ no AM / SM light beige (RAL1001)	++ no AM / SM copper brown (RAL8004) / exopigment	+++ warm ivory AM/ SM sand yellow (RAL1002	+++ no AM / SM ochre yellow (RAL1024)	+ no AM / SM beige (RAL1001)	++ no AM / SM ochre yellow (RAL1024)

+, poor growth; ++, moderate growth; +++ good growth; SM, substrate mycelium; AM, aerial mycelium.

Supplementary Material

Table S2. Fatty acid profile of the strains DSM 116494^T and DSM 116496^T and their close phylogenomic relatives *Streptomyces pilosus* DSM 40097^T, *Streptomyces griseoflavus* DSM 40456^T, and *Streptomyces vinaceus* DSM 40515^T.

	**Studied strain**	**Close phylogenetic relatives**		**Studied strain**	**Close phylogenetic relative**
**Fatty acids**	**Isolate DSM 116494^T**	***S. griseoflavus* DSM 40456^T**	***S. pilosus* DSM 40097^T**	**Isolate DSM 116496^T**	***S. vinaceus* DSM 40515^T**
$C_{12:0}$	0.1	NA	0.1	0.1	0.1
iso-$C_{13:0}$	0.2	NA	0.2	0.3	0.4
anteiso-$C_{13:0}$	0.2	0.1	0.2	0.2	0.2
$C_{13:0}$	NA	NA	NA	0.1	NA
iso -$C_{14:0}$	2.4	3.7	1.4	2.1	2.4
$C_{14:0}$	0.7	0.8	0.6	1.4	1.0
iso-$C_{15:0}$	7.3	3.8	7.0	11.0	9.9
anteiso-$C_{15:0}$	22.3	19.8	13.1	27.5	29.1
$C_{15:1}$ cis 9	0.3	0.4	0.3	0.2	0.1
$C_{15:0}$	3.0	4.1	2.1	4.0	2.1
iso-$C_{16:1}$ cis 9	2.4	3.5	1.9	0.4	0.3
iso -$C_{16:0}$	16.9	23.2	16.6	8.1	9.4
$C_{16:1}$ cis 7	NA	NA	NA	0.1	NA
$C_{16:1}$ cis 9	8.8	9.6	7.7	7.0	3.9
$C_{16:0}$	9.6	9.2	8.0	18.9	15.8
iso-$C_{17:1}$ cis 9	3.0	1.3	5.7	1.2	1.1
anteiso-$C_{17:1}$ cis 9	4.0	4.1	5.0	1.0	1.3
iso-$C_{17:0}$	2.6	1.2	5.6	3.9	6.4
anteiso-$C_{17:0}$	12.3	10.2	18.7	8.4	12.4
$C_{17:1}$ cis 9	1.6	2.2	2.1	0.9	0.5
$C_{17:1}$ cis 11	0.2	0.4	NA	NA	NA
$C_{17:0}$ cyclo cis 9	0.1	NA	0.3	0.9	1.1
$C_{17:0}$	1.0	1.0	1.3	1.5	1.2
iso -$C_{18:1}$ cis 9	0.2	0.3	0.5	NA	NA
iso -$C_{18:1}$ cis 11	0.2	0.4	0.3	NA	NA
anteiso -$C_{18:1}$ cis 9	NA	NA	0.3	NA	NA
iso -$C_{18:0}$	0.1	0.1	NA	0.1	0.2
$C_{18:1}$ cis 9	0.3	0.2	0.4	0.3	0.2
$C_{18:1}$ cis 11	0.1	0.3	0.3	0.2	0.1
$C_{18:1}$ trans 11	NA	NA	NA	NA	0.1
$C_{18:0}$	0.2	NA	0.2	0.3	0.5
iso -$C_{19:0}$	NA	NA	NA	NA	0.1
anteiso -$C_{19:0}$	NA	NA	0.1	NA	NA

NA, not available.

Supplementary Material

Table S3. Pairwise 16S rRNA gene sequence similarity between the strains DSM 116494^T and DSM 116496^T and their closest phylogenetic neighbours.

Isolates	Species validly named	Strain designation	Accession numbers	Pairwise Similarity (%)
DSM 116494 ^{T}	*Streptomyces marokkonensis*	Ap1^T	AJ965470	99.4
	Streptomyces rubrogriseus	LMG 20318^T	AJ781373	99.4
	Streptomyces althioticus	NRRL B-3981^T	AY999791	99.4
	Streptomyces malachitofuscus	NBRC 13059^T	AB184282	99.3
	Streptomyces lienomycini	LMG 20091^T	AJ781353	99.3
	Streptomyces thinghirensis	DSM 41919^T	FM202482	99.2
	Streptomyces griseoflavus	LMG 19344^T	AJ781322	99.2
	Streptomyces violaceoruber	NBRC 14892	AB184631	99.2
	Streptomyces violaceoruber	NBRC 12826^T	AB184174	99.2
	Streptomyces violaceoruber	NBRC 15461	AB184687	99.2
	Streptomyces ambofaciens	ATCC 23877^T	CP012382	99.1
	Streptomyces tendae	ATCC 19812^T	D63873	99.1
	Streptomyces violaceorubidus	LMG 20319^T	AJ781374	99.1
	Streptomyces griseoloalbus	NBRC 13046^T	AB184275	99.0
	Streptomyces albaduncus	JCM 4715^T	AY999757	99.0
	Streptomyces heliomycini	NBRC 15899^T	AB184712	99.0
	Streptomyces djakartensis	NBRC 15409^T	AB184657	98.9
	Streptomyces tritolerans	DAS 165^T	DQ345779	98.9
	Streptomyces paradoxus	NBRC 14887^T	AB184628	98.9
	Streptomyces viridochromogenes	NBRC 3113^T	AB184728	98.9
	Streptomyces tuirus	NBRC 15617^T	AB184690	98.9
	Streptomyces albogriseolus	NRRL B-1305^T	AJ494865	98.8
	Streptomyces griseoincarnatus	LMG 19316^T	AJ781321	98.8
DSM 116496^T	*Streptomyces xanthophaeus*	NRRL B-5414^T	JOFT01000080	99.7

Streptomyces cirratus	NRRL B-3250^{T}	AY999794	99.7
Streptomyces nojiriensis	LMG 20094^{T}	AJ781355	99.7
Streptomyces sporoaraveus	LMG 20313^{T}	AJ781370	99.7
Streptomyces subrutilus	DSM 40445^{T}	X80825	99.7
Streptomyces avidinii	NBRC 13429^{T}	AB184395	99.7
Streptomyces vinaceus	NBRC 13425^{T}	AB184394	99.7
Streptomyces lavendulae subsp. *lavendulae*	NRRL B-2774^{T}	JOEW01000098	99.6
Streptomyces goshikiensis	NBRC 15458	AB184684	99.6
Streptomyces goshikiensis	NBRC 12868^{T}	AB184204	99.6
Streptomyces netropsis	NRRL B-1990	DQ026646	99.5
Streptomyces virginiae	NRRL ISP-5094^{T}	JOAK01000082	99.4
Streptomyces manipurensis	MBRL 201^{T}	JN560156	99.2
Streptomyces spiroverticillatus	NBRC 12821^{T}	AB249921	98.8
Streptomyces adustus	WH-9^{T}	LC026279	98.8
Streptomyces cavourensis	NBRC 13026^{T}	AB184264	98.7
Streptomyces griseus	NRRL ISP-5322	MUNB01000146	98.7

Table S4. Digital DNA-DNA hybridization between the whole genome sequence of strains DSM 116494^{T} and DSM 116946^{T} and their close phylogenomic neighbours.

**Isolates**	**Close phylogenetic neighbours**	**dDDH (d4, in %)**
Strain DSM 116494^{T}	*Streptomyces griseoflavus* JCM 4479^{T}	39.0
Strain DSM 116494^{T}	*Streptomyces malachitofuscus* JCM 4493^{T}	38.6
Strain DSM 116494^{T}	*Streptomyces flavoviridis* JCM 4372^{T}	38.5
Strain DSM 116494^{T}	*Streptomyces pilosus* JCM 4403^{T}	38.5
Strain DSM 116494^{T}	*Streptomyces atrovirens* JCM 6913^{T}	37.3
Strain DSM 116494^{T}	*Streptomyces griseoloalbus* JCM 4480^{T}	36.2
Strain DSM 116494^{T}	*Streptomyces albaduncus* JCM 4715^{T}	36.1
Strain DSM 116494^{T}	*Streptomyces capillispiralis* DSM 41695^{T}	35.5
Strain DSM 116494^{T}	*Streptomyces capillispiralis* JCM 5075^{T}	35.3
Strain DSM 116494^{T}	*Streptomyces aureorectus* DSM 41692^{T}	34.6
Strain DSM 116494^{T}	*Streptomyces virens* JCM 9095^{T}	34.5
Strain DSM 116494^{T}	*Streptomyces asterosporus* DSM 41452^{T}	34.4
Strain DSM 116494^{T}	*Streptomyces calvus* T-3018^{T}	34.4

Strain DSM 116494^{T}	*Streptomyces calvus* JCM 4326^{T}	34.3
Strain DSM 116494^{T}	*Streptomyces griseomycini* JCM 4382^{T}	34.0
Strain DSM 116494^{T}	*Streptomyces griseostramineus* JCM 4385^{T}	33.9
Strain DSM 116494^{T}	*Streptomyces ghanaensis* ATCC 14672^{T}	33.3
Strain DSM 116494^{T}	*Streptomyces albogriseolus* JCM 4616^{T}	31.5
Strain DSM 116494^{T}	*Streptomyces albogriseolus* JCM 4536	31.5
Strain DSM 116496^{T}	"*Streptomyces kutzneri*" DSM 40907^{T}	29.4
Strain DSM 116496^{T}	*Streptomyces vinaceus* ATCC 27476^{T}	29.4
Strain DSM 116496^{T}	*Streptomyces goshikiensis* JCM 4640^{T}	29.3
Strain DSM 116496^{T}	*Streptomyces nojiriensis* JCM 3382^{T}	29.2
Strain DSM 116496^{T}	*Streptomyces spororaveus* NBRC 15456^{T}	29.1
Strain DSM 116496^{T}	*Streptomyces subrutilus* ATCC 27467^{T}	29.0
Strain DSM 116496^{T}	*Streptomyces xanthophaeus* NRRL B-5414^{T}	29.0
Strain DSM 116496^{T}	*Streptomyces avidinii* DSM 40526^{T}	28.9
Strain DSM 116496^{T}	*Streptomyces avidinii* JCM 4726^{T}	28.9
Strain DSM 116496^{T}	*Streptomyces lavendulae* subsp. *grasserius* JCM 4056^{T}	28.7
Strain DSM 116496^{T}	*Streptomyces virginiae* NRRL ISP-5094^{T}	28.7
Strain DSM 116494^{T}	Strain DSM 116496^{T}	22.3

Table S5. Antimicrobial bioassay of strains DSM 116494^{T} and DSM 116496^{T} against Gram-positive (multi-resistant *Staphylococcus aureus* DSM 18827 and *Enterococcus faecium* DSM 20477^{T}) and Gram-negative bacteria (*Escherichia coli ΔtolC* JW5503-1, and *Proteus vulgaris* DSM 2140), as well as yeast (*Candida albicans* DSM 1386). The values in the table correspond to the diameter of the inhibition zone (in mm).

		reference strains				
**Ethyl acetate extracts**	**Media**	***E. coli ΔtolC*** JW5503-1	***P. vulgaris*** DSM 2140	***S. aureus*** DSM 18827	***E. faecium*** DSM 20477^{T}	***C. albicans*** DSM 1386
Strain DSM 116494^{T}	NL19	9	-	-	-	-
Strain DSM 116494^{T}	NL800	8	-	-	-	-
Strain DSM 116494^{T}	R5	7	-	28	-	-
Strain DSM 116496^{T}	NL19	7	-	-	-	-
Strain DSM 116496^{T}	NL800	8	-	-	-	-

Supplementary Material

Table S6. BCGs of secondary metabolites for the isolates DSM 116494^{T} and DSM 116496^{T} and their closest phylogenomic neighbours using the antiSMASH server.

			**DSM 116494^{T}**	***S. griseoflavus* JCM 4479^{T}**	***S. pilosus* JCM 4403^{T}**	**DSM 116496^{T}**	***S. vinaceus* ATCC 27476^{T}**
**Type**	**Most similar known cluster**		**Similarity (%)**				
PKS-like,NRPS,other	A54145	NRP		15	-	8	8
hydrogen-cyanide,lanthipeptide-class-iii	aborycin	RiPP	64	57	-	21	21
betalactone,T1PKS	meilingmycin	Polyketide	6	-	-	-	
butyrolactone	lactonamycin	Polyketide	-	-	-	3	
butyrolactone	rubiginone A2/rubiginone J/rubiginone K/rubiginone L/rubiginone M/rubiginone N/ochromycinone/rubiginone B2	Polyketide	-	-	-		10
butyrolactone	triacsin C	Other	-	-	-	6	
CDPS	ibomycin	Polyketide	-	-	-	4	
CDPS	tetronasin	Polyketide	-	-	-	3	
ectoine	ectoine	Other	100	100	100	100	

furan,butyrolactone	methylenomycin A	Other	-	23%	-		
hglE-KS,T1PKS	hexacosalactone A	Other	-	-	-	6	9
hydrogen-cyanide,lanthipeptide-class-iii	SapB	RiPP:Lanthipeptide	-	-	100	100	100
lanthipeptide-class-i,NRPS,lanthipeptide-class-ii	teicoplanin	NRP:Glycopeptide	5	-	-	-	
lanthipeptide-class-iii,HR-T2PKS,thioamitides,arylpolyene,T2PKS,butyrolactone	prejadomycin/rabelomycin/gaudimycin C/gaudimycin D/UWM6/gaudimycin A	Polyketide:Type II polyketide+Saccharide:Hybrid/tailoring saccharide	-	-	-	43	
lanthipeptide-class-iv	accramycin A	Polyketide		-	-		5
lanthipeptide-class-iv	corbomycin	NRP	-	-	-	7	
LAP	streptamidine	RiPP:Other	-	100	100		
LAP,butyrolactone	esmeraldin	Polyketide+Other:Aminocoumarin	-	-	8		
LAP,thiopeptide,RRE-containing	streptovaricin	Polyketide	17	-	-	-	
lassopeptide	vazabitide A	NRP	-	-	-		13
lassopeptide,lanthipeptide-class-i	citrulassin B	RiPP	-	-	-	40	
linaridin	pentostatine/vidarabine	Other		-	-		9
melanin	melanin	Other	-	-	42	100	28
NAPAA	ε-Poly-L-lysine	NRP	-	-	-	100	100
NI-siderophore	desferrioxamin B/desferrioxamine E	Other	100	100	100		100
NI-siderophore	kinamycin	Polyketide	16	13	19	-	13
NI-siderophore	paulomycin	Other	11	11	11	-	-

NRP-metallophore,NRPS	peucechelin	NRP	50	-	-	-	-
NRPS	cinnapeptin	NRP	-	-	-	14	-
NRPS	tirandamycin	NRP+Polyketide:Modular type I polyketide	-	66	-		-
NRPS,betalactone	enduracidin	NRP	33	-	-	20	-
NRPS,butyrolactone	coelichelin	NRP	-	-	-	100	-
NRPS,lanthipeptide-class-i,lanthipeptide-class-ii,RiPP-like	jagaricin	NRP	-	-	-		13
NRPS,NRPS-like	zorbamycin	NRP:Glycopeptide+Polyketide: Modular type I polyketide+Saccharide:Hybrid/tailoring saccharide	-	-	91		
NRPS,NRPS-like,blactam	lipopeptide 8D1-1/lipopeptide 8D1-2	NRP	-	-	-		4%
NRPS,T1PKS	antimycin	NRP+Polyketide	-	-	93		
NRPS,terpene	depsibosamycin B/depsibosamycin C/depsibosamycin D	NRP	-	-	-		14
NRPS-like	conglobatin	NRP	-	-	-	15	-
NRPS-like	streptozotocin	Other	23	-	-	-	-
NRPS-like,NRPS	antipain	NRP		-	100	-	-

NRPS-like,NRPS	capreomycin IA/capreomycin IB/capreomycin IIA/capreomycin IIB	NRP		9	-	-	-
NRPS-like,NRPS	friulimicin A/friulimicin B/friulimicin C/friulimicin D	NRP		-	75	-	-
NRPS-like,T1PKS	amipurimycin	Polyketide+NRP+Saccharide		-	96	-	-
NRPS-like,T1PKS	borrelidin	Polyketide	11	-	-	-	-
NRPS-like,T1PKS	miharamycin B/miharamycin A	Polyketide+NRP+Saccharide		-	-	-	89
NRPS-like,T1PKS,butyrolactone	neocarzinostatin	Polyketide:Iterative type I polyketide+Polyketide:Enediyne type I polyketide	-	52	-	-	-
other	lidamycin	NRP+Polyketide	-	25	-	-	-
phenazine	endophenazine A/endophenazine B	Other:Phenazine	-	-	-	38	-
PKS-like	trioxacarcin A	Polyketide	-	-	7		-
PKS-like,NRPS	cadaside A/cadaside B	NRP	19	-	-	-	-
resorcinol	lasalocid	Polyketide	11	-	-	-	-
RiPP-like	granaticin	Polyketide:Type II polyketide	16	-	8		8
RiPP-like	informatipeptin	RiPP:Lanthipeptide	42	42	42		-
RRE-containing	oryzanaphthopyran A/oryzanaphthopyran B/oryzanaphthopyran C/oryzanthrone A/oryzanthrone	Polyketide	6	-		-	-

	B/chlororyzanthrone A/chlororyzanthrone B						
T1PKS,lanthipeptide-class-i,NRPS,NAPAA	stenothricin	NRP:Cyclic depsipeptide	-	-		13	-
T2PKS	spore pigment	Polyketide	83	83	83	66	-
T2PKS,T1PKS	homopiloquinone/piloquinone	Polyketide	-	-	100		-
T3PKS	alkylresorcinol	Polyketide	-	-	-		100
T3PKS,NI-siderophore	kinamycin	Polyketide	-	-	-	30	-
T3PKS,other,terpene	furaquinocin B	Terpene+Polyketide	-	-	-	39	-
terpene	albaflavenone	Terpene	100	100	100	-	-
terpene	avermitilol	Terpene	-	-	-	-	100
terpene	desotamide	NRP	13%	-	-	-	-
terpene	geosmin	Terpene	100	100	100	100	-
terpene	hopene	Terpene	92%	92%	92%	61%	61%
terpene	isorenieratene	Terpene	100	87	87	-	-
terpene	monensin	Polyketide	-	-	-	5	5
terpene	oxalomycin B	NRP+Polyketide	-	-	-	-	12
terpene	toxoflavin/fervenulin	Other	-	-	-	-	14
terpene,NRPS	azomycin	Other	-	-	-	83	-
terpene,T3PKS	flaviolin/1,3,6,8-tetrahydroxynaphthalene	Polyketide	-	-	-	-	100
thiopeptide,LAP	lactazole	RiPP:Thiopeptide	-	-	-	-	66

Supplementary Material
